# Increase of the Insane, and Some Problems Connected with Their Public Care

**Published:** 1887-12

**Authors:** William D. Granger

**Affiliations:** First Assistant Physician, Buffalo State Asylum for the Insane, Buffalo, N. Y.


### Selections.

### *INCREASE OF THE INSANE, AND SOME PROBLEMS CONNECTED WITH THEIR PUBLIC CARE.*

By WILLIAM D. GRANGER, M. D.,

First Assistant Physician, Buffalo State Asylum for the Insane, Buffalo, N. Y.

There is much talk about the rapid increase of insanity, and but little exact knowledge in the public mind regarding it. It is easy, from the records of this State, to show just what the visible increase is. What is true here, is practically true of the whole country. By visible insane I mean all who are in asylums.

From October 1, 1880, to October 1, 1886, there has been an increase of 4,001 insane persons in the various asylums in this state, or an average of 666 yearly. The increase for 1886 was 831. In 1870, there were 5,673 insane persons in our state, county, city and private asylums; in 1880, there were 9,537, and in 1886, they had increased to 13,538. But a small part of these are supported at private expense; they are mostly a public charge. The same rate of increase would give 16,202 to be cared for in 1890, and 22,862 in 1900.

A little further study will show that the insane are increasing in a more rapid ratio than the population. In 1870, there was one insane person in the various asylums of the state to every 779 of the population; in 1880, one in every 532; and in 1886, one in every 406. This last is based upon an estimated population of 5,503,543, in accordance with the known increase from 1870 to 1880. Probably, the population is some greater and the proportion a little too high.

While an increase of 666 insane, scattered over the State of New York, is easily assimilated, yet, continued year after year, it makes an increase difficult to care for, and a demand upon state, county and city.

What a task the state has before it, alone, in caring for this class of its dependent citizens! It must yearly provide for over 600 new, though by no means recent, cases of insanity. It is easy to illustrate what this means. The State Asylum at Utica has a capacity for 600 patients. It reports the value of its buildings and farm to be $670,822. The yearly increase of the insane is greater than the capacity of the Utica Asylum, and if they are to be cared for, as they are in that institution, it means an annual expenditure of over $700,000. And, practically, unless the insane are to be improperly cared for, and crowded into the county and city asylums, this increase cannot be met by an expenditure of less than $500,000 a year. So that by the year 1900, the state, counties and cities will be called upon to expend $7,000,000 for this object. Will they do it? Probably they will not, but they *must* do a large part of it.

It is not within the province of this paper to discuss the causes for this increase. It is a very complex and not well-understood subject. There are, however, a few causes easily observed, aside from the increase of the population.

1. The large number of foreigners coming into our state, that are either insane, or possess in themselves, or their first generation, all the essential elements to produce the disease.

2. The prolonged life of the chronic insane, due to their improved care in the many asylums of the State.

3. The improper and delayed treatment in too many of the county asylums, or in the homes of the insane, during the acute and curable stage.

4. The increase of asylums, and their nearness to the people, makes them more used.

Of the first cause, there can be no reason to look for its limitation of action without efficient laws and active control by the General

Government. There is a limit to the action of the second cause, and in the older countries, like England, it is thought to have been already reached. Nothing but strong legislative action, and a watchful Supervisory State Commission with ample powers, can control the third cause. While there are many notable exceptions, too many counties, from false ideas of economy, send their acute insane to their county asylums, or refuse to furnish public aid to send them to state institutions. The fourth cause is in active operation, and, by fixed laws, always exerts an influence in increasing the number of the insane supported at public charge.

It is safe to say that the number of our insane will increase until one in less than every 400 of our population will be confined in our state, county, city and private asylums.

It is very much to be regretted, that the reports of our asylums do not furnish that information by which two very important questions can be answered: First, are the number of acute and curable cases of insanity increasing in a ratio greater than the increase of population, and to what extent do they add to the chronic class? And, second, are those types of insanity which, from the beginning, belong to the chronic and incurable class, increasing in a like ratio?

This lesson of the past should be the guide of the state in its future policy. It should be a wise, humane, economical and settled policy. It should make unstinted efforts to stay this increase, and should afford every means to promote the recovery or improve the condition of each individual insane person. It should rigidly require of those public officials of our counties, cities and towns, into whose hands all of the indigent and pauper insane first come, to immediately provide for their being brought under such care and treatment as each case shall need.

It should even be remembered that many of the chronic insane may be so benefitted by treatment as to be able to return to their homes, perhaps to help to support their family or themselves, or at least relieve the public from their support.

